# Sorafenib- Taurine Combination Model for Hepatocellular Carcinoma Cells: Immunological Aspects 

**DOI:** 10.31557/APJCP.2019.20.10.3007

**Published:** 2019

**Authors:** Ahmed M Afifi, Ahmed M El-Husseiny, Reda H Tabashy, Mohamed A Khalil, Motawa E El-Houseini

**Affiliations:** 1 *Department of Zoology, Faculty of Science, *; 2 *Department of Cancer Biology, *; 3 *Department of Diagnostic Radiology, *; 4 *Department of Clinical Pathology, National Cancer Institute, Cairo University, Cairo, Egypt. *

**Keywords:** HCC, Sorafenib, Taurine, FOXP3, cytokines

## Abstract

Sorafenib (Sor) is a multi-kinase inhibitor. It is recommended for the treatment of advanced hepatocellular carcinoma (HCC). However, Sor has severe and marked side effects. On the other hand, taurine (Tau) has been shown to enhance the therapeutic effects of cancer chemotherapy and also to enhance the function of leukocytes.

Here, we aimed to investigate the enhancing efficacy of Sor as well as minimizing its marked side effects by using Tau in combination in an immunological aspect. We evaluated the influence of Sor and Tau combination on the expression pattern of FOXP3 gene in HepG2 cells compared to peripheral blood mononuclear leukocytes (PBMCs) as control normal cells. Also, the levels of TGF-β and IL-10 released in culture media of both cells were determined. Our results revealed that, Tau reduced cytotoxicity of Sor on PBMC indicated by lactic dehyrogenase (LDH) release assay. In addition, Sor-Tau combination led to FOXP3 down-regulation in hepatic cancer cells (HepG2). The results showed also that, TGF-β levels decreased significantly in their culture media. In contrary, the cytokine increased in PBMCs culture media. Moreover, IL-10 was significantly elevated in the culture media of both cells. This study could open new avenues for the improvement of therapeutic efficacy of Sorafenib treated HCC patients by using Tau in combination.

## Introduction

Hepatocellular carcinoma (HCC) is one of the leading causes of cancer-related deaths worldwide (Allaire and Nault, 2017). Sorafenib (Sor), a multi-kinase inhibitor which targeting tumor angiogenesis induces apoptosis was approved by US FDA for the treatment of advanced HCC (Ou et al., 2010; Bruix and Sherman, 2011). However, only around 30% of patients will be benefited with Sor, and will show resistance often within 6 months. Furthermore, there is no effective therapy available after failure of Sor treatment (Llovet et al., 2008; Kim et al., 2017). Moreover, Sorafenib has a cardiotoxic effects results from alterations of myocyte calcium homeostasis (Schneider et al., 2018). 

Taurine (Tau), is an amino acid derivative in human body, exists in various kinds of cells and exerts multiple biological actions. It regulates intracellular calcium homeostasis, maintain cell membrane stability and protect cells (Inoue et al., 2012; De Luca et al., 2015; Prentice et al., 2015; Čolović et al., 2017). Indeed, currently taurine has been approved for heart failure treatment (Schaffer and Kim, 2018). Regarding to our interest in the immune system, Tau is rich in neutrophilic granulocytes, lymphocytes and monocytes, accounting for 50%–70% of the total free amino acids. Where it has been shown that Tau enhances the function and inhibits the apoptosis of leukocytes, and may be of therapeutic potential in minimizing the systemic side effects of chemotherapeutics (Çetiner et al., 2005; Wang et al., 2009). Also, several studies have demonstrated the antitumor activity of Tau against cancer cells (Zhang et al., 2014; Tu et al., 2015; Zhang et al., 2015; El Houseini et al., 2017).

Combination therapies strategy one of the promising approaches for cancer therapy. In a study compared the metabolite composition profile of HepG2 cells treated with sorafenib-everolimus combination therapy showed that sorafenib therapy preferentially targets glycerophospholipid and purine metabolism, while everolimus affects pyruvate, amino acid, and glucose metabolism in HCC cells (Zheng et al., 2015). We here used another model of combination to study its effect on hepatoma cells in term of immunological aspects.

Forkhead BoxP3 (FOXP3) is a key transcription factor in T regulatory cells (T-regs), and its role in immunosuppressive functions (Hori and Sakaguchi, 2004). Prevalence of T-regs (CD4+CD25+FOXP3+) in peripheral blood and tumor infiltrating of HCC patients lead to an unfavorable prognostic, promote disease progression and may prevent effective antitumor immune responses (Ormandy et al., 2005; Fu et al., 2007; Kobayashi et al., 2007; Mathai et al., 2012). Also, it has been shown that, cancer cells express FOXP3, suggesting that FOXP3 may have some roles in cancer progression (Lu, 2008; Fontenot et al., 2005; Martin et al., 2010). It was reported that, FOXP3 expression in some hepatoma cell lines and HCC sections revealed that 48% of HCC displayed FOXP3 staining, but FOXP3 staining were absent in normal liver tissues and para-tumor tissues. Therefore, it is worth exploring the function and role of FOXP3 in the cirrhosis and development of HCC (Chen et al., 2008; Wang et al., 2010). Moreover; FOXP3 expression has crucial role in regulation of several cytokines, such as transforming growth factor-β (TGF-β) and interleukin-10 (IL-10). Also, FOXP3 might mediate the inhibiting efficacy of tumor cells to escape immune attack (Wang et al., 2010). 

TGF-β is a regulatory cytokine with various functions, including cell proliferation, differentiation, and extracellular matrix remodeling (Chruścik et al., 2017). It exerts tumor-suppressive effects against cancer cells. Paradoxically, TGF-β also modulates some processes such as cell invasion, immune regulation, and microenvironment modification in tumor development when the signaling pathway is mis-regulated (Massagué, 2008). Also, TGF-β is over produced by tissue leading to elevation in plasma levels of patients with HCC (Teicher, 2001). Furthermore; Inhibitors of the TGF-β signaling have been shown to block HCC growth and progression by modulating epithelial-mesenchymal transition (EMT) (Giannelli et al., 2014). It has been shown also that, there are two different TGF-β signaling responses, one called an “early” and another termed “late” TGF-β signaling response (Coulouarn et al., 2008). For instance, the early response pattern is associated with longer and the late response pattern with shorter survival (Coulouarn et al., 2008). It is possible that the early response pattern reflects the physiologic inflammatory response, while the late response is associated with a long-term TGF-β activation (Calon et al., 2013). 

IL-10 is a cytokine known for its immune-regulatory activity on antigen- presenting cells (APCs), T cells, and other immune cells (Wang et al., 2017). Also, IL-10 contributes to poor prognosis, worse tumor staging and low anti-tumor immunity in patients with unresectable HCC (Hattori et al., 2003; Shin et al., 2003; Chan et al., 2012). It may also reflect the degree of inflammation (Shimizu et al., 2012). In addition, tumors from non-responder mice showed more abundance of Foxp3+ regulatory T cells and IL-10 than responders to cancer chemotherapy such as doxorubicin administration (Zabala et al., 2007). Whereas, Inflammatory cytokines and chronic inflammation correlate with increased tumor incidence and a worsened prognosis for patients with malignancies (Coussens et al., 2013). Moreover, IL-10 has anti-inflammatory and pro-immunity functions which inhibits tumor promoting inflammation and induces CD8+ cells. Recombinant human IL-10 induced the expression of IFN-γ and granzymes in tumor-bearing mice through CD8‏+ T-cell–dependent manner (Berman et al., 1996; Oft, 2014). 

Our study aims to reduce the cytotoxicity of Sor on PBMCs by Tau using LDH release assay. Also, to investigate “Sor-Tau” combination effect on FOXP3 gene expression and its intracellular staining by flowcytometry in cancer cells as well as PBMCs as normal control cells. Moreover, to evaluate the levels of TGF-β and IL-10 released in culture media of HepG2 cells compared to PBMCs before and after treatment with sorafenib-Taurine combination model.

## Materials and Methods

*HepG2 cells and PBMCs Isolation*

HCC cell line HepG2 was obtained from the American Type Culture Collection (ATCC, Minisota, USA). The tumor cell line maintained at National Cancer Institute (NCI), Cairo, Egypt. Peripheral blood was obtained from HCC patients enrolled at NCI, Cairo, Egypt. Peripheral blood mononuclear cells (PBMCs) were isolated using density gradient with Ficoll (Biowest, France). The cells were grown in RPMI-1640 Medium (Biowest, France) supplemented with 10% Fetal Bovine Serum (FBS) (Biowest, France), Sodium Biocarbonate 7.5% (Biowest, France), 100 U/ml penicillin and 100 mg/ml streptomycin (Biowest, France) in 5% CO_2_ at 37°C.

*Compounds*

- Two hundred milligram (200 mg) of Sor (Nexavar tablet, Byern, Germany) was dissolved in 31.4 mL 100% DMSO, stored as primary stock 10 mmol at -4 °C and diluted in RPMI to the required concentration with DMSO concentration > 0.1.

- Eighty milligram (80 mg) of Taurine powder (Bio Basic INC, Canada) was dissolved in 1 ml RPMI-1640 medium, equivalent to 400 mM as primary stock and diluted to required concentration.

*Cytotoxicity Assay*

*SRB Assay*

HepG2 cells (5-10×10^3^ cells/well) were plated onto 96-well microplates. They were incubated overnight in 5% CO_2_ at 37°C. Cells were divided into untreated cells as a control or treated ones with Sor, Tau and in combination of Sor-Tau for 48 h. at different concentrations. Cytotoxicity was measured using the SRB assay (SIGMA Life science, USA), which is based on measurements of cellular protein content according to the Nature Protocol (Vichai and Kirtikara, 2006). Absorbance was measured at 570 nm using a microplate reader (Tecan, Sunrise, Austria). All assays were performed in triplicates.

*Lactate Dehydrogenase (LDH) Measurements*

PBMCs were isolated from HCC patients. They were treated with 5µM Sor, 160mM Tau and in combination for 48 h compared to control untreated cells. Culture media were collected for measuring LDH activity via a chemical colorimetric method (Spectrum).

*Flow Cytometric Analysis*

The Cells was permeabolized with Tween 20 plus PBS and fixed with 0.5% paraformaldehyde then the staining with intracellular Anti-Human Foxp3 PE-Cyanine7 (eBioscience, Affymetrix, San Diego, CA, USA) performed according to manufacturer’s instruction, they were detected by BD FACS flow cytometer (BD, Franklin lakes, NJ, USA).

*Real-Time RT–PCR Analysis*

Total RNA was isolated from hepG2 cell line, PBMCs exposed to Sor and Tau and in combination using Rneasy mini kit (QIAGEN) and was used as a template for the synthesis of complementary DNA by using High-Capacity cDNA Archive Kit (Applied Biosystems). Quantitative qPCR (Via7) using SYBR GREEN (QuantiTect^®^ SYBR^®^ Green PCR, QIAGEN) was carried out using FOXP3 (forward primer) 5’- CAG CAC ATT CCC AGA GTT CCT C -3’ and FOXP3 (reverse primer) 5’- GCG TGT GAA CCA GTG GTA GAT C -3’. PCR involved 40 cycles of 95 ˚C for 15 sec, 58 ˚C for 30 sec, and 72˚C for 30 sec, with a final Melt Curve cycle 95˚C 15 sec, 58˚C 1 min. The 2^-∆∆Ct^ method was used to calculate values of FoxP3 fold change relative to β-Actin gene amplification.

*Cytokine Measurements*

IL-10 (BMS215/2, Affymetrix, eBioscience) levels and TGF-β (KOMA BIOTECH INC., Korea) levels were measured in Culture media of untreated and treated cells with Sor, Tau and combination for 48 h by ELISA according to the manufacturer’s direction.

*Statistical Analysis*

All experiments were repeated three times and were conducted in triplicate. Data are expressed as the Mean ± SD. and tested for statistically significance by Graph prism version 5 using one way analysis of variance (ANOVA) followed by Turkey Crammer test for post-hoc analysis, at p<0.05.

## Results

*Proliferation of HepG2 cells*

**Figure 1:**
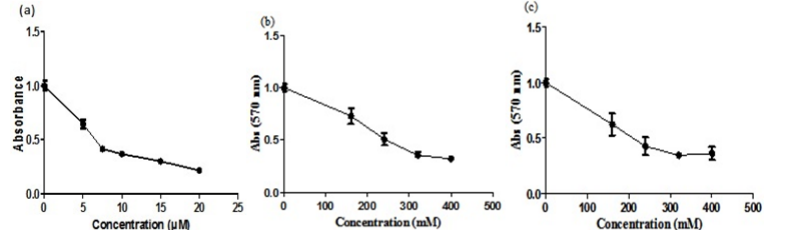
Determination of HepG2 Cells Vability Using SRB Assay. HepG2 cells were treated with Sor (a), Tau (b) or their combination (c) at the indicated concentrations. In (c) cells were treated with fixed dose of Sor (5µM) and different doses of Tau (as indicated). Graphs show the mean value **±** SD. Experiment was performed three times, and each value was read in triplicate

HepG2 cells were treated for 48h with several doses of Sor (5, 10, 15, 20 μM), Tau (160, 240, 320, 400 mM) and Combination (5 μM of Sor with 160-400 mM of Tau) (Figure 1). The IC50 concentration was 7.21 **±** 0.60 μM, 220.67 **±** 27.5 mM and 198.7 **±** 25.7 mM for Sor, Tau and both respectively. 

*LDH activity in PBMC culture media of PBMC*

**Figure 2:**
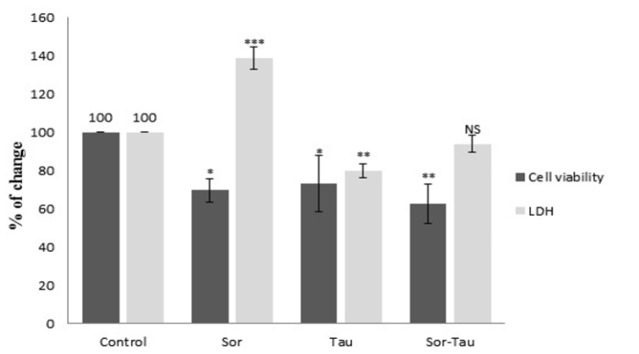
Cell Viability of HepG2 Cells versus LDH Activity Levels in Culture Media of PBMCs. Sorafenib (5µM), Taurine(160mM) and in Combination (5µM,160mM) showed a significant inhibition of HepG2 cells proliferation for 48 h. Cell viability calculated by [O.D (treated cells)/ O.D (control cells) %]. The corresponded activity of LDH released in media at the same doses in PBMCs were measured. Values represented as Mean **±** Standard Deviation, Significantly different from control at p<0.05*, p<0.001**, p<0.0001***.

LDH activity released in culture media of PBMCs of Sor-treated group recording 82.5**±**1.54 IU/L as compared to control untreated (59.5**±**0.85 IU/L). While, LDH activity in Tau-treated group was (47.5**±**0.22 IU/L) and in combination was (55.8**±**0.8 IU/L). Percentage of change of LDH activity in culture media of PBMC was calculated and compared to cell viability of HepG2 cells (Figure 2).

*FOXP3 expression in HepG2 cells and PBMC*

**Figure 3:**
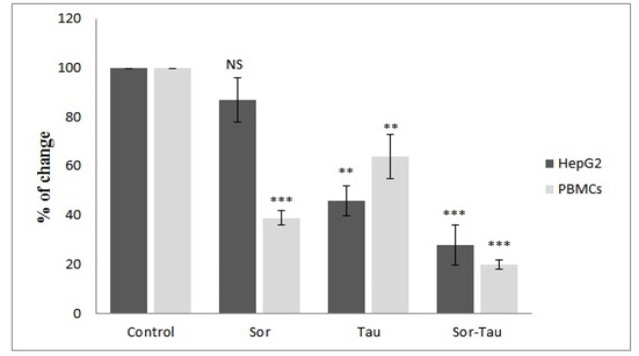
FOXP3 Gene Expression. Real time PCR of FOXP3 gene expressions fold change. FOXP3 gene expression in HepG2 cells and PBMCs after sorafenib, taurine and in combination compared to control-untreated group. Values represented as Mean**±**SD, Significantly different from control at p<0.001**, p<0.0001***. Fold Change equals 2^-∆∆Ct^.

FOXP3 expression was determined at the mRNA and protein levels. First, mRNA quantitation indicated that all drug treatments down-regulated FOXP3 mRNA in HepG2 cells and PBMCs. For instance, Sor inhibited FOXP3 mRNA in 40% and 87% in PBMCs HepG2 cells, respectively. Tau suppressed FOXP3 mRNA expression 64% and 46% in PBMC and HepG2, respectively. Also, the combination affected mRNA of FOXP3 in PBMC as well as HepG2 cells recording 20% and 28% respectively (Figure 3).

**Figure 4:**
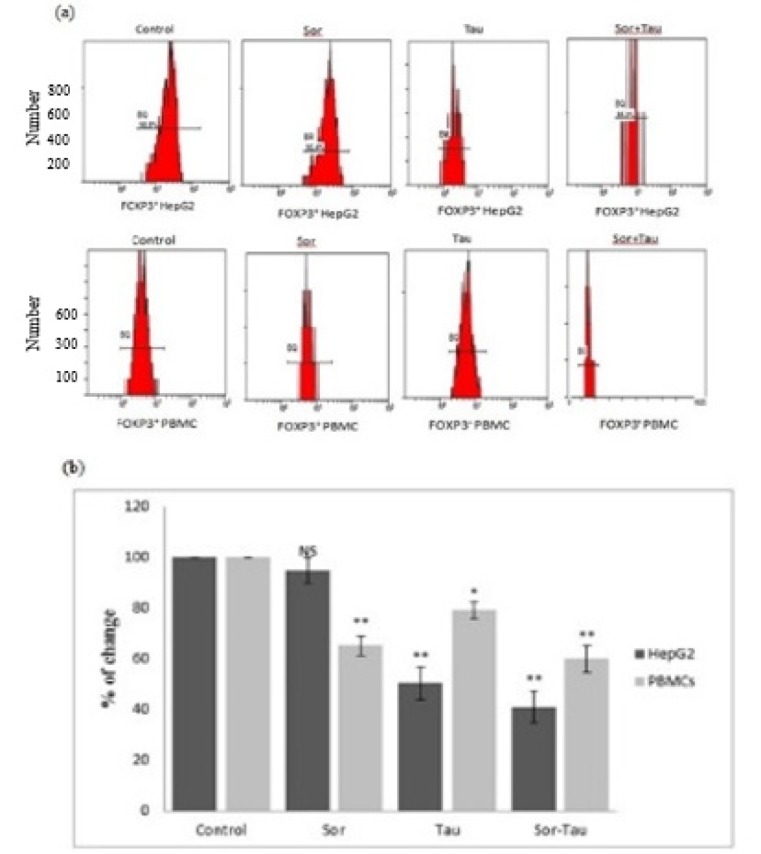
Flowcytometric analysis of intracellular staining of FOXP3. (a): Data represents the effect of Sor, Tau and in combination on FOXP3 in HepG2 cells and PBMCs. (b): Results represent Mean**±** SD of x-mean value percentage from flowcytometric analysis, significantly different from control at p<0.05*, p<0.001**, p<0.0001***.

Intracellular staining of FOXP3 (Figure 4) confirmed the above gene expression results where, the percentages of change in controls were 100%. Data obtained from Sor groups were 95% in HepG2 cells and 65% in PBMCs. By using Tau alone, the percentages were significantly reduced in both groups to 50% and 79%. After “Sor-Tau” combination the percentage of change to control exhibited 41% and 60%.

**Table 1:** TGF-β Levels in Culture Media of HepG2 and PBMCs Treated with Sorafenib, Taurine and in Combination

Groups	HepG2 (GA)			PBMC (GB)		
	TGF-β (Pg/ml)	P-value	% of change§	TGF-β (Pg/ml)	P-value	% of change§
	Mean ± SD			Mean ± SD		
1- Control (Untreated)	1936.3 ± 23.1	-	100%	403.6 ± 48.4	-	100%
2- Sorafenib	149.1 ± 10.1	< 0.0001	7.70%	473 ± 89.9	NS	117%
3- Taurine	1473.5 ± 33.3	< 0.0001	76.10%	975.8 ± 112	< 0.05	241.80%
4- "Sor and Tau"	313 ± 18.5	< 0.0001	16.20%	1037.4 ± 98.2	< 0.001	257%

Values represented as Mean**±** SD, Significantly different from control at p<0.05, p<0.0001. §, % of change is calculated in relation to control.

**Table 2:** IL-10 Levels in Culture Media of HepG2 and PBMCs Treated with Sorafenib, Taurine and in Combination

Groups	HepG2 (GA)			PBMC (GB)		
	IL-10 (Pg/ml) Mean ± SD	P-value	% of Change§	IL-10 (Pg/ml) Mean ± SD	P-value	% of Change§
1- Control (Untreated)	2.03 ± 0.39	-	100%	2.22 ± 0.4	-	100%
2- Sorafenib	18.7 ± 0.98	< 0.0001	921.20%	11.8 ± 0.48	< 0.0001	531.50%
3- Taurine	18.1 ± 1.10	< 0.0001	891.60%	12.8 ± 0.84	< 0.0001	576.60%
4- "Sor and Tau"	72.6 ± 0.74	< 0.0001	3576.30%	48.9 ± 1.2	< 0.0001	2202.70%

Values represented as Mean **±**SD, Significantly different from control at *p<0.05, p<0.0001*. §, % of change is calculated in relation to control.

*TGF-β and IL-10 measurements*

The level of TGF-β decreased significantly in culture media of HepG2 cells after treatment with Sor recording 149.1 ± 10.1 Pg/ml compared to the untreated ones. In case of cells treated with Tau the cytokine level was recording 1473.5**±** 33.3 Pg/ml. As for Sor-Tau combination, the value obtained was 313 **± **18.5 Pg/ml compared to control untreated culture media of cancer cells that was recording 1936.3 **±** 23.1 Pg/ml. On the contrary, elevated levels in the cytokine in PBMC culture media were observed in Sor treated cells that was recording 473 **± **89.9 Pg/ml and in case of Tau treated ones, the recorded level raised to 975.8 **± **112 Pg/ml, while the elevation level was highly significant in case of “Sor-Tau”-treated cells that was recording 1,037.4 **± **98.2 Pg/ml compared to control untreated culture media of PBMCs (Table 1). 

Table 2 summarized that; IL-10 levels were recording 2.03 **± **0.39, 2.22 ±0.4 in culture media of both HepG2 and PBMC cells, respectively. While, after treatment with Sor an increase of the interleukin was observed in culture media of HepG2 and PBMC cells which were recording 18.7 **±**0.98 Pg/ml and 11.8 **± **0.48 Pg/ml, respectively. Similarly, in Tau-treated cells, the recorded values were 18.1 **± **1.10 Pg/ml and 12.8 **± **0.84 Pg/ml in culture media of both cells respectively. Whereas, there were highly significant elevations of IL-10 in culture media of “Sor-Tau” combination treated HepG2 and PBMCs cells which were recording 72.6 **± **0.74 Pg/ml and 48.9 **± **1.2 Pg/ml, respectively.

## Discussion

It has been shown that, HCC has a high mortality and recurrence rates and Sorafenib (Sor) is the first multi-kinase inhibitor used to treat the advanced stage of the disease (Bertino, 2012). Treated patients usually suffer from severe side effects, developing a rapid resistance and the median survival is only 3 months (Shimizu et al., 2012). Therefore, searching for ancillary drugs which can enhance the therapeutic effects and overcome the adverse effects of cancer chemotherapy is strongly recommended (Wang et al., 2009). 

On the other hand, it has been shown that, Taurine (Tau) is closely correlated with immune function where it is highly abundant in leukocytes (Schuller-Levis and Park, 2004; Maher et al., 2005). Additionally, Taurine induces apoptosis in human cancer cells such as colon, hepatocellular carcinoma HepG2 and breast cancer cells (Zhang et al., 2014; Tu et al., 2015; Zhang et al., 2015).

Our current study revealed that, Combination of Sor and Tau showed a significant inhibition of cell growth of HepG2 cells growing in culture compared to those treated with Sor alone. In addition, Tau significantly reduced the cytotoxic effect of Sor on mononuclear leukocytes isolated from patients suffering from HCC indicated by LDH levels released in their culture media. Our data is in agreement with an experimental animal model where, Tau enhanced the function of the leucocytes after chemotherapy in tumor-bearing mice (Wang et al., 2009).

We found that, “Sor-Tau” Combination led to down-regulation of FOXP3 expression in both PBMC and HepG2 cells elucidated by mRNA levels and Flowcytometric analysis. The results revealed that, Sor treated cells alone showed a significant higher expression of FOXP3 gene in PBMC than that of HepG2 cells, and vice versa in case of Tau treated cells which reduced the FOXP3 gene expression in HepG2 cells more significantly than that in PBMC. These data indicated that, FOXP3 gene could be useful criteria for Sor and Tau efficacy. 

Several studies have pointed out the influence of Sor on FOXP3 gene expressed in TILs or Tregs (Cabrera et al., 2013; Kalathil et al., 2013; Wang et al., 2013). However, as a matter of fact, our work is the first in our knowledge to study the effect of Sor-Tau combination on FOXP3 gene expression in HCC cells propagated ex-vivo compared to PBMCs of HCC patients.

Furthermore, we found also that, TGF-β levels have different correspond in culture media of HepG2 cells and PBMCs where they decreased and increased respectively, due to treatment with Sor or Tau alone and in combination compared to control untreated cells. 

Herein, regarding IL-10 results; the present work showed its elevation in culture media of both HepG2 and PBMCs after exposure to Sor and Tau and recorded the highest value after their Combination. “Gopinathan et al., (2017); Chung et al., (2017)” reported that, the increasing levels of IL-10 in culture media of PBMCs treated cells could be attributed to the role of IL-10 in controlling tumor cells promoting inflammation. Furthermore, in another study They suggested that the release of IL-10 levels in both serum and ascites is one of the adaptive resistance mechanism that reflects the efficacy of anti-PD-1 or anti-PD L1 monotherapies (Lamichhane et al., 2017). 

Finally, the main objective of the present work is to evaluate “Sor-Tau” combination therapy against HepG2 cells in comparison with PBMC of HCC patients in term of immunological aspect. Further investigation is strongly recommended to use “Sor-Tau” combination as a potential useful effective therapy for the treatment of patients suffering from HCC.
